# Dietary supplement use for eye health in adults: knowledge, motivations, and consumption patterns in a nationwide sample of adults in Poland, 2026

**DOI:** 10.3389/fnut.2026.1866810

**Published:** 2026-06-15

**Authors:** Agnieszka Kamińska, Radosław Sierpiński, Agata Olearczyk, Maciej Kamiński, Mariusz Gujski, Mateusz Jankowski, Grzegorz Juszczyk

**Affiliations:** 1 Faculty of Medicine, Collegium Medicum, Cardinal Stefan Wyszynski University, Warsaw, Poland; 2 National Centre for Health Policy and Health Inequalities, Cardinal Stefan Wyszynski University, Warsaw, Poland; 3 Faculty of Medicine, Łazarski University, Warsaw, Poland; 4 VIZJA University, Warsaw, Poland; 5 School of Public Health, Centre of Postgraduate Medical Education, Warsaw, Poland; 6 Department of Public Health, Faculty of Health Sciences, Medical University of Warsaw, Warsaw, Poland

**Keywords:** adult population, cross-sectional study, dietary supplements, eye health, health behavior, nutrition knowledge, Poland

## Abstract

**Introduction:**

Vision is a key human sense affecting both social and occupational activities. Evidence on the role of nutrients and micronutrients in eye health remains inconsistent. This study aimed to assess self-reported knowledge of diet and eye health and to characterize the use of dietary supplements for vision support among adults in Poland.

**Methods:**

This cross-sectional questionnaire-based survey was conducted between 13 and 17 March 2026 on a nationwide sample of 1,090 adults in Poland. Self-reported questionnaire was used. Data were collected using Computer-Assisted Web Interviews (CAWI).

**Results:**

Most respondents reported low or moderate self-reported knowledge of the impact of diet and lifestyle on eye health, with only 10.2% declaring good knowledge. Among the respondents, 27.9% declared use dietary supplements for eye health. Among users, supplementation was mainly driven by preventive decisions (38.8%) and medical recommendations (28.0%), with antioxidant vitamins and omega-3 fatty acids being the most commonly used ingredients. Female gender (aOR = 1.51; 95%CI:1.01–2.27) and residence in both small (<20,000) and large (≥500,000) cities (aOR = 2.76 and 3.01, respectively) were associated with higher odds of reporting good knowledge. Active occupational status (aOR:1.91; 95%CI:1.33–2.75; *p* < 0.001), wearing spectacles or contact lenses (aOR:1.76; 95%CI:1.22–2.53; *p* = 0.002), and history of eye conditions diagnosed by a doctor (aOR:3.25; 95%CI:2.41–4.38; *p* < 0.001) were significantly associated with higher odds of using dietary supplements for eye health in the past 12 months.

**Conclusion:**

This study shows that dietary supplement use for eye health is relatively common among adults in Poland despite low self-reported knowledge on diet and vision. Supplement use was more frequent among individuals with eye conditions, those wearing corrective lenses, and occupationally active participants, and was not fully aligned with self-reported knowledge. These findings highlight the need for improved public education and stronger involvement of healthcare professionals in promoting evidence-based supplement use.

## Introduction

Vision is a key human sense affecting both social and occupational activities (1, 2). The World Health Organization (WHO) estimates that 2.2 billion people worldwide have near or distance vision impairment (3). Population aging, growing adoption of digital technologies and lifestyle-changed significantly contribute to increasing burden of eye conditions (4–6). The most common eye conditions such as age-related macular degeneration (AMD), cataracts, glaucoma, and dry eye syndrome are determined by multiple factors, including genetic, environmental and lifestyle-related factors (5, 7, 8). Diet is an important modifiable lifestyle-related risk factor contributing to eye health (9, 10).

While certain micronutrients, such as vitamin A, are essential for maintaining normal visual function, evidence regarding the effectiveness of dietary supplements and nutraceuticals in preventing, slowing, or reversing eye diseases remains limited and inconsistent (9–14). However, growing evidence suggests that specific nutrients such as lutein, zeaxanthin, omega-3 fatty acids, zinc and selected antioxidants may contribute to eye health, and may help prevent or slow the progression of certain eye conditions (15–17). In response to the growing public interest in maintaining eye health through lifestyle-related interventions such as diet, the market for vision-supporting dietary supplements has expanded (13, 14, 18). The widespread promotion of dietary supplements supporting eye health and vision has led to increased visibility and uptake among the general population (19).

Despite the steady expansion of the market for nutraceuticals targeting eye health, scientific evidence on their effectiveness, especially in primary prevention of eye conditions remains inconsistent or even inconclusive (11–18). Effectiveness of nutraceuticals in ophthalmology is condition-specific and stage-dependent, and is mostly limited to tertiary prevention of AMD (15, 16). The Age-Related Eye Disease Study (AREDS) and its subsequent modification, AREDS2, demonstrated that specific antioxidant formulations may reduce the risk of progression from intermediate to advanced age-related macular degeneration (AMD) by approximately 25% in high-risk individuals (15). The currently recommended AREDS2 formulation includes vitamin C, vitamin E, zinc, copper, lutein, and zeaxanthin, while beta carotene present in the original AREDS formulation was removed due to its association with increased lung cancer risk among smokers (15). Scientific evidence on the effectiveness of supplementation with antioxidant vitamins C/E or zinc solely, as opposed to combined formulations like AREDS, remains limited (15, 16). Lutein and zeaxanthin may delay progression of AMD and cataract, but their role in disease prevention remains unclear (16, 20). Omega-3 fatty acids, including docosahexaenoic acid (DHA) and eicosapentaenoic acid (EPA) may support retinal health and improve dry eye symptoms due to their anti-inflammatory activity and role in tear film stabilization (17, 21).

Despite limited scientific evidence on the impact of dietary supplements on eye health in general adults, these products are widely marketed (15–21). Consequently, the use of dietary supplements for eye health may be driven not only by medical recommendations but also by individual behaviors, perceptions, marketing and non-verified sources of information (22, 23).

Public awareness of the impact of diet and lifestyle on eye health may shape consumer choices and individual behaviors related to dietary supplement use (6, 12, 22, 24). Previous studies have indicated that knowledge gaps may coexist with relatively high levels of supplement use, suggesting a potential mismatch between evidence-based recommendations and real-world behaviors (25–27). This phenomenon may be observed particularly in countries with high availability of dietary supplements, relatively permissive regulatory frameworks, and strong influence of marketing and non-professional information sources, which may further contribute to self-directed and potentially suboptimal health decisions (19, 28, 29).

The dietary supplement market in Poland has expanded rapidly in recent years and is one of the most dynamic segments of the health-related consumer sector (19, 30). Its value exceeded 7 billion Polish zloty PLN (1.6 billion EUR) in 2024, with forecast indicating further growth in the coming years (19, 30). When compared to other European Union (EU) countries, Poland is characterized by rapid market growth, high over-the-counter availability and high consumption of dietary supplements (19, 29, 30). Recent evidence from nationally representative studies in Poland indicates that dietary supplement use is highly prevalent, with approximately 39% of adults reporting regular use and an additional 31.5% occasional use within a 3-month period (31). Notably, only a minority of users rely exclusively on medical recommendations, as approximately 36% report consulting a physician regarding supplement use (31). These findings highlight that, despite widespread consumption, dietary supplements are frequently used as part of self-directed health practices rather than under professional supervision.

As dietary supplements are frequently used as part of self-care practices rather than based on professional recommendations, self-perceived knowledge of diet and its role in eye health, including the role of dietary supplementation may play important role in shaping health-related behaviors (25, 26, 32). Understanding how individuals perceive their knowledge, what motivates supplement use, and which ingredients are most commonly consumed is essential from a public health perspective. Moreover, these data may help identify populations at risk of misinformation, unnecessary supplementation, or suboptimal dietary practices. However, there is a lack of nationwide data on the use of dietary supplements specifically aimed at supporting eye health among adults in Poland.

Therefore, this study aimed to evaluate self-reported knowledge of diet in relation to eye health and to characterize the use of dietary supplement use for vision support in a nationwide sample of adults in Poland.

## Materials and methods

### Study design and population

This cross-sectional questionnaire-based survey was conducted between 13 and 17 March 2026 on a nationwide sample of adults in Poland. Data were collected using Computer-Assisted Web Interview (CAWI) method. A dedicated public opinion survey company (Nationwide Research Panel Ariadna) (33) was contracted for data collection. All procedures and methodological assumptions were provided by the authors.

Participants were recruited from a panel comprising over 100,000 unique and verified individuals (33). A quota sampling was applied, with stratification model based on gender, age and location of the place of residence. The sampling framework was aligned with national demographic distributions as expressed in the Statistics Poland demographic reports (34). As 96% of households in Poland has an Internet access, the study sample was considered representative of the adults population (34).

Inclusion criteria were Polish nationality, age ≥18 years, and ability to complete the questionnaire in Polish. Individuals younger than 18 years or unable to complete the questionnaire were excluded from participation.

Eligible participants were invited via email and text messages (33). Each participant received a unique URL link to the online questionnaire hosted on a professional survey platform managed by the Nationwide Research Panel Ariadna (33). If the respondent refused to participate in the study, the next respondent who matched the stratification criteria was selected. There were no missing answers. In total, 1,090 fully completed questionnaires were included in the analysis.

Similar data collection procedures were applied in previously published studies based on nationwide samples of adults in Poland (35, 36).

### Study questionnaire and measures

The study questionnaire included 16 questions on eye health, dietary supplement use, and preventive behaviors and was developed based on a targeted literature review focusing on nutrition, dietary supplementation, and eye health (10–18). The questionnaire content and thematic scope were developed jointly by the study authors, including researchers with experience in public health, epidemiology, and health behavior research. The final version of the questionnaire was established following discussion and consensus among all authors.

Self-reported knowledge of the impact of diet and lifestyle on vision and eye health was measured using a five-point Likert scale (very low/none, low, moderate, rather good, very good). For statistical analysis, responses “rather good” or “very good” were combined into one category.

Participants were also asked about the use of dietary supplements or vitamin–mineral preparations intended to support eye health or vision within the past 12 months with the following question: During the past 12 months, have you used any dietary supplements or vitamin–mineral preparations intended to support eye health or vision?, and six possible answers: “I did not use any,” “I used them for no longer than 1 month,” “I used them for 2–6 months,” “I used them for 7–12 months,” and “I do not remember.” In this analysis, respondents who declared “I used them for no longer than 1 month,” “I used them for 2–6 months,” “I used them for 7–12 months” were classified as those who used dietary supplements or vitamin–mineral preparations intended to support eye health or vision within the past 12 months. Among participants who reported supplement use, reasons for use were assessed using a multiple-response question, including medical recommendations, pharmacist advice, self-initiated preventive use, presence of symptoms, and external influences (e.g., advertising, social media, or recommendations from family and friends).

All participants were asked about the ingredients included in the dietary supplements or vitamin–mineral preparations you used in the past 12 months (consumed event without intention related to eye health). Information on the composition of supplements was collected using a multiple-response question, including lutein or zeaxanthin, omega-3 fatty acids (DHA/EPA), zinc, antioxidant vitamins (e.g., vitamin C or E), and multicomponent formulations for eye health. Respondents could also indicate other ingredients.

A pilot study was conducted among 18 adults, who completed the questionnaire twice with a 7 day interval. Following this procedure, four items were revised to improve readability and consistency. Kappa coefficients for the critical questions ranged from 0.64 to 0.92.

### Statistical analysis

Analyses were performed using IBM SPSS Statistics, version 29 (IBM Corp., Armonk, NY, United States). Categorical variables were presented with frequencies and proportions. Associations between respondents’ socio-demographic characteristics and self-reported knowledge or dietary supplement use were assessed with cross-tabulations and chi-square tests. Two different logistic regression models were prepared to identify factors associated with (1) self-reported knowledge of the impact of diet and lifestyle on vision and eye health and (2) use of any dietary supplements or vitamin–mineral preparations intended to support eye health or vision in the past 12 months. In the first step, each sociodemographic variable (11 different independent variables) was analyzed separately using bivariable logistic regression. Variables significant in bivariable models (*p* < 0.05) as well as variables considered epidemiologically or clinically relevant based on previous literature were entered into the multivariable logistic regression models. Associations were presented with odds ratios (ORs) along with 95% confidence intervals (CIs). Statistical significance level was set at *p* < 0.05.

## Results

The study included 1,090 respondents, of whom 54.1% were female (Table 1). The largest age group comprised individuals aged ≥60 years (29.6%). Most participants had secondary (42.7%) or higher education (45.9%) and were married (53.8%). The majority were occupationally active (61.5%) and reported good or moderate financial status (85.7%). Additionally, 69.3% used spectacles or contact lenses, and 35.4% had a diagnosed eye condition. Details are presented in Table 1.

**Table 1:** Characteristics of the study population (*n* = 1,090).

Variable	*n*	%
Gender		
Female	590	54.1
Male	500	45.9
Age [years]		
18–29	139	12.8
30–39	220	20.2
40–49	205	18.8
50–59	203	18.6
60+	323	29.6
Educational level		
Primary or vocational	125	11.5
Secondary	465	42.7
Higher	500	45.9
Marital status		
Single	285	26.1
Married	586	53.8
Informal relationship	161	14.8
Other	58	5.3
Place of residence		
Rural area	432	39.6
City below 20,000 residents	142	13.0
City from 20,000 to 99,999 residents	207	19.0
City from 100,000 to 499,999 residents	171	15.7
City ≥ 500,000 residents	138	12.7
Having children under 18 years		
Yes	275	25.2
No	815	74.8
Number of household members		
1 (living alone)	178	16.3
2	397	36.4
3 or more	515	47.2
Occupational status		
Active	670	61.5
Passive	420	38.5
Self-declared financial status		
Good	510	46.8
Moderate	424	38.9
Bad	156	14.3
Wearing spectacles or contact lenses		
Yes	755	69.3
No	335	30.7
Eye condition diagnosed by a doctor		
Yes	386	35.4
No	704	64.6

Most respondents reported moderate (34.5%) or low levels of knowledge regarding the impact of diet and lifestyle on vision and eye health, with only 10.2% declaring rather good or very good knowledge (Table 2). Over half of the participants (59.1%) did not use any dietary supplements intended to support eye health in the past 12 months. Among users, supplementation was most commonly reported for short periods (≤6 months). Among supplement users (*n* = 304), the most common reasons for use were personal preventive decisions (38.8%), recommendations from an ophthalmologist (28.0%), and the presence of symptoms (25.3%). Among all respondents (*n* = 1,090), the most frequently reported ingredients included antioxidant vitamins (21.4%) and omega-3 fatty acids (20.6%), followed by zinc (13.5%) and lutein or zeaxanthin (12.7%) (Table 2).

**Table 2:** Public knowledge of diet and lifestyle in relation to eye health and dietary supplement use for vision support in a nationwide sample of adults (*n* = 1,090).

Variable	*n*	%
How would you assess your knowledge of the impact of diet and lifestyle on vision and eye health?		
Very low level of knowledge or lack of knowledge	298	27.3
Low level of knowledge	304	27.9
Moderate level of knowledge	376	34.5
Rather good level of knowledge	93	8.5
Very good level of knowledge	19	1.7
What ingredients were included in the dietary supplements or vitamin–mineral preparations you used in the past 12 months? (multiple-response question)		
I did not use any supplements or preparations during this period	483	44.3
Lutein or zeaxanthin	138	12.7
Omega-3 fatty acids (DHA/EPA)	225	20.6
Zinc	147	13.5
Antioxidant vitamins (e.g., vitamin C or E)	233	21.4
Multicomponent eye health formulations (marketed or recommended for vision)	64	5.9
Other ingredients supporting eye health or vision (please specify)	12	1.1
I do not remember the ingredients of the supplements or preparations used	164	15.0
During the past 12 months, have you used any dietary supplements or vitamin–mineral preparations intended to support eye health or vision?		
I did not use any	644	59.1
I used them for no longer than 1 month	103	9.4
I used them for 2–6 months	113	10.4
I used them for 7–12 months	88	8.1
I do not remember	142	13.0
For what reasons did you use dietary supplements or vitamin–mineral preparations intended to support eye health or vision? (*n* = 304) (multiple-response question)		
Recommendation of an ophthalmologist	85	28.0
Recommendation of a general practitioner or another specialist (non-ophthalmologist)	54	17.8
Pharmacist’s advice	36	11.8
Own preventive decision (as a precaution)	118	38.8
Due to symptoms (e.g., dry eyes or eye fatigue)	77	25.3
Influenced by advertising	19	6.3
Influenced by opinions on social media	30	9.9
Recommendation from family or friends	39	12.8
Other reason (please specify)	3	1.0

Self-reported knowledge of the impact of diet and lifestyle on vision and eye health varied (*p* < 0.05) by gender, educational level, and place of residence (Table 3). In multivariable logistic regression model, female gender (aOR:1.51; 95%CI:1.01–2.27; *p* = 0.04) and living in cities below 20,000 residents (aOR:2.76; 95%CI:1.53–4.98; *p* < 0.001) or cities ≥ 500.000 residents (aOR:3.01; 95%CI:1.68–5.39; *p* < 0.001) were significantly associated with higher odds of reporting very good or rather good knowledge of the impact of diet and lifestyle on vision and eye health (Table 3). The explanatory performance of the multivariable logistic regression model was modest, which is expected in population-based analyses of self-reported behavioral and knowledge-related outcomes. The pseudo-R^2^ values were 0.021 according to Cox & Snell, 0.044 according to Nagelkerke, and 0.032 according to McFadden.

**Table 3:** Factors associated with very good or rather good self-reported knowledge of the impact of diet and lifestyle on vision and eye health (*n* = 1,090).

			Bivariable logistic regression		Multivariable logistic regression	
Variable	%	*p*	OR (95%CI)	*p*	aOR (95%CI)	*p*
How would you assess your knowledge of the impact of diet and lifestyle on vision and eye health?—“very good” or “rather good” level of knowledge						
Gender						
Female (*n* = 590)	12.0	**0.04**	1.53 (1.02–2.30)	0.04	1.51 (1.01–2.27)	**0.04**
Male (*n* = 500)	8.2		Reference		Reference	
Age [years]						
18–29 (*n* = 139)	12.9	0.4	1.22 (0.67–2.25)	0.5		
30–39 (*n* = 220)	10.9		1.01 (0.58–1.75)	0.9		
40–49 (*n* = 205)	10.2		0.94 (0.53–1.66)	0.8		
50–59 (*n* = 203)	6.9		0.61 (0.32–1.16)	0.1		
60+ (*n* = 323)	10.8		Reference			
Educational level						
Primary or vocational (*n* = 125)	7.2	**0.004**	Reference			
Secondary (*n* = 465)	7.5		1.05 (0.49–2.25)	0.9		
Higher (*n* = 500)	13.6		2.03 (0.98–4.19)	0.06		
Marital status						
Single (*n* = 285)	10.9	0.7	Reference			
Married (*n* = 586)	9.4		0.85 (0.53–1.35)	0.5		
Informal relationship (*n* = 161)	12.4		1.16 (0.64–2.12)	0.6		
Other (*n* = 58)	10.3		0.95 (0.38–2.38)	0.9		
Place of residence						
Rural area (*n* = 432)	6.5	**<0.001**	Reference		Reference	
City below 20,000 residents (*n* = 142)	16.2		2.79 (1.55–5.02)	<0.001	2.76 (1.53–4.98)	**<0.001**
City from 20,000 to 99,999 residents (*n* = 207)	8.7		1.37 (0.74–2.55)	0.3	1.36 (0.73–2.52)	0.3
City from 100,000 to 499,999 residents (*n* = 171)	11.1		1.80 (0.98–3.32)	0.06	1.79 (0.97–3.30)	0.06
city ≥500,000 residents (*n* = 138)	17.4		3.04 (1.70–5.44)	<0.001	3.01 (1.68–5.39)	**<0.001**
Having children under 18 years						
Yes (*n* = 275)	8.0	0.2	0.70 (0.43–1.14)	0.2		
No (*n* = 815)	11.0		Reference			
Number of household members						
1 (living alone) (*n* = 178)	10.7	0.7	Reference			
2 (*n* = 397)	11.1		1.04 (0.59–1.84)	0.9		
3 or more (*n* = 515)	9.5		0.88 (0.50–1.54)	0.7		
Occupational status						
Active (*n* = 670)	11.0	0.3	1.25 (0.83–1.88)	0.3		
Passive (*n* = 420)	9.0		Reference			
Self-declared financial status						
Good (*n* = 510)	12.5	0.07	1.58 (0.85–2.95)	0.2		
Moderate (*n* = 424)	8.3		0.99 (0.51–1.92)	0.9		
Bad (*n* = 156)	8.3		Reference			
Wearing spectacles or contact lenses						
Yes (*n* = 755)	9.7	0.3	0.81 (0.54–1.23)	0.3		
No (*n* = 335)	11.6		Reference			
Eye condition diagnosed by a doctor						
Yes (*n* = 386)	10.9	0.6	1.11 (0.74–1.66)	0.6		
No (*n* = 704)	9.9		Reference			

Statistically significant values are bolded.

Use of any dietary supplements or vitamin–mineral preparations intended to support eye health or vision in the past 12 months varied (*p* < 0.05) by gender, age, educational level, occupational status, wearing spectacles or contact lenses and history of eye condition diagnosed by a doctor (Table 4). In multivariable logistic regression model, active occupational status (aOR:1.91; 95%CI:1.33–2.75; *p* < 0.001), wearing spectacles or contact lenses (aOR:1.76; 95%CI:1.22–2.53; *p* = 0.002), and history of eye conditions diagnosed by a doctor (aOR:3.25; 95%CI:2.41–4.38; *p* < 0.001) were significantly associated with higher odds of using dietary supplements or vitamin–mineral preparations intended to support eye health or vision in the past 12 months (Table 4). In contrast, age 50–59 years (aOR:0.60; 95%CI:0.38–0.96; *p* = 0.03) was significantly associated with lower odds of supplement use. The explanatory performance of the multivariable logistic regression model was modest but acceptable for a population-based behavioral model, with Cox & Snell R^2^ of 0.111 and Nagelkerke R^2^ of 0.160.

**Table 4:** Factors associated with the use of any dietary supplements or vitamin–mineral preparations intended to support eye health or vision in the past 12 months (*n* = 1,090).

			Bivariable logistic regression		Multivariable logistic regression	
Variable	%	*p*	OR (95%CI)	*p*	aOR (95%CI)	*p*
Use of any dietary supplements or vitamin–mineral preparations intended to support eye health or vision in the past 12 months						
Gender						
Female (*n* = 590)	30.8	**0.02**	1.38 (1.06–1.81)	0.02	1.30 (0.97–1.73)	0.08
Male (*n* = 500)	24.4		Reference		Reference	
Age [years]						
18–29 (*n* = 139)	38.1	**0.004**	1.46 (0.96–2.21)	0.08	1.57 (0.96–2.56)	0.07
30–39 (*n* = 220)	29.5		0.99 (0.68–1.44)	0.9	0.91 (0.57–1.45)	0.7
40–49 (*n* = 205)	21.0		0.63 (0.42–0.95)	0.03	0.63 (0.38–1.04)	0.07
50–59 (*n* = 203)	23.2		0.71 (0.48–1.07)	0.1	0.60 (0.38–0.96)	**0.03**
60+ (*n* = 323)	29.7		Reference		Reference	
Educational level						
Primary or vocational (*n* = 125)	16.8	**0.01**	Reference			
Secondary (*n* = 465)	29.2		2.05 (1.23–3.41)	0.05		
Higher (*n* = 500)	29.4		2.06 (1.24–3.42)	0.06		
Marital status						
Single (*n* = 285)	27.4	0.9	Reference			
Married (*n* = 586)	27.5		1.01 (0.73–1.38)	0.9		
Informal relationship (*n* = 161)	30.4		1.16 (0.76–1.78)	0.5		
Other (*n* = 58)	27.6		1.01 (0.54–1.90)	0.9		
Place of residence						
Rural area (*n* = 432)	26.4	0.7	Reference			
City below 20,000 residents (*n* = 142)	31.0		1.25 (0.83–1.90)	0.3		
City from 20,000 to 99,999 residents (*n* = 207)	30.9		1.25 (0.87–1.80)	0.2		
City from 100,000 to 499,999 residents (*n* = 171)	26.3		1.00 (0.67–1.49)	0.9		
City ≥ 500,000 residents (*n* = 138)	26.8		1.02 (0.66–1.58)	0.9		
Having children under 18 years						
Yes (*n* = 275)	27.6	0.9	0.98 (0.73–1.33)	0.9		
No (*n* = 815)	28.0		Reference			
Number of household members						
1 (living alone) (*n* = 178)	25.3	0.4	Reference			
2 (*n* = 397)	26.7		1.08 (0.72–1.61)	0.7		
3 or more (*n* = 515)	29.7		1.25 (0.85–1.84)	0.3		
Occupational status						
Active (*n* = 670)	30.3	**0.03**	1.37 (1.04–1.81)	0.03	1.91 (1.33–2.75)	**<0.001**
Passive (*n* = 420)	24.0		Reference		Reference	
Self-declared financial status						
Good (*n* = 510)	28.0	0.5	1.21 (0.80–1.83)	0.4		
Moderate (*n* = 424)	29.0		1.27 (0.83–1.93)	0.3		
Bad (*n* = 156)	24.4		Reference			
Wearing spectacles or contact lenses						
Yes (*n* = 755)	32.8	**<0.001**	2.44 (1.76–3.37)	<0.001	1.76 (1.22–2.53)	**0.002**
No (*n* = 335)	16.7		Reference		Reference	
Eye condition diagnosed by a doctor						
Yes (*n* = 386)	45.3	**<0.001**	3.70 (2.80–4.88)	<0.001	3.25 (2.41–4.38)	**<0.001**
No (*n* = 704)	18.3		Reference		Reference	

Statistically significant values are bolded.

The use of specific ingredients in dietary supplements varied across socio-demographic groups. Females more frequently reported the use of lutein or zeaxanthin and omega-3 fatty acids compared to males (*p* < 0.05). Omega-3 fatty acids were most commonly used among individuals aged 30–39 years (30.9%; *p* < 0.001), and their use also differed by educational level, with the highest prevalence among those with higher education (25.4%; *p* < 0.001). The use of lutein or zeaxanthin varied by place of residence (*p* = 0.02). Additionally, individuals wearing spectacles or contact lenses and those with a diagnosed eye condition were significantly more likely to report the use of most ingredients, including lutein or zeaxanthin, omega-3 fatty acids, antioxidant vitamins, and multicomponent formulations (*p* < 0.05). Details are presented in Table 5.

**Table 5:** Sociodemographic differences in composition of dietary supplements and vitamin–mineral preparations used in the past 12 months (*n* = 1,090).

	Lutein or zeaxanthin		Omega-3 fatty acids (DHA/EPA)		Zinc		Antioxidant vitamins (e.g., vitamin C or E)		Multicomponent eye health formulations	
Variable	%	*p*	%	*p*	%	*p*	%	*p*	%	*p*
What ingredients were included in the dietary supplements or vitamin–mineral preparations you used in the past 12 months? (multiple-response question)										
Gender										
Female (*n* = 590)	15.4	**0.003**	23.4	**0.02**	14.4	0.3	23.4	0.08	7.5	**0.02**
Male (*n* = 500)	9.4		17.4		12.4		19.0		4.0	
Age [years]										
18–29 (*n* = 139)	12.2	0.06	16.5	**<0.001**	17.3	0.3	23.7	0.1	4.3	0.9
30–39 (*n* = 220)	11.8		30.9		11.4		26.8		6.4	
40–49 (*n* = 205)	7.3		14.1		10.2		17.1		6.3	
50–59 (*n* = 203)	13.8		18.7		14.3		22.2		5.4	
60+ (*n* = 323)	16.1		20.7		14.9		18.9		6.2	
Educational level										
Primary or vocational (*n* = 125)	3.2	**0.003**	12.8	**<0.001**	8.8	0.1	16.0	0.1	2.4	0.2
Secondary (*n* = 465)	14.0		17.6		12.7		20.2		6.2	
Higher (*n* = 500)	13.8		25.4		15.4		23.8		6.4	
Marital status										
Single (*n* = 285)	10.5	0.4	18.2	**0.02**	12.3	0.7	22.1	0.7	4.2	0.5
Married (*n* = 586)	13.5		20.1		14.0		20.6		6.1	
Informal relationship (*n* = 161)	11.8		29.2		14.9		24.2		7.5	
Other (*n* = 58)	17.2		13.8		10.3		17.2		6.9	
Place of residence										
Rural area (*n* = 432)	8.6	**0.02**	17.4	0.3	11.1	0.2	20.6	0.8	4.6	0.4
City below 20,000 residents (*n* = 142)	14.8		22.5		14.1		23.9		6.3	
City from 20,000 to 99,999 residents (*n* = 207)	15.0		23.7		14.5		21.7		5.8	
City from 100,000 to 499,999 residents (*n* = 171)	14.0		21.1		13.5		22.8		5.8	
City ≥ 500,000 residents (*n* = 138)	18.1		23.9		18.8		18.8		9.4	
Having children under 18 years										
Yes (*n* = 275)	12.7	0.9	21.8	0.6	13.1	0.8	22.9	0.5	6.9	0.4
No (*n* = 815)	12.6		20.2		13.6		20.9		5.5	
Number of household members										
1 (living alone) (*n* = 178)	14.6	0.7	15.2	0.09	11.2	0.07	16.3	0.2	5.6	0.8
2 (*n* = 397)	12.3		23.2		16.6		21.7		5.3	
3 or more (*n* = 515)	12.2		20.6		11.8		22.9		6.4	
Occupational status										
Active (*n* = 670)	13.3	0.4	22.4	0.07	13.4	0.9	23.7	**0.02**	6.6	0.2
Passive (*n* = 420)	11.7		17.9		13.6		17.6		4.8	
Self-declared financial status										
Good (*n* = 510)	12.2	0.3	21.4	0.8	11.8	0.3	20.2	0.5	5.1	**0.03**
Moderate (*n* = 424)	14.4		20.3		15.1		23.1		8.0	
Bad (*n* = 156)	9.6		19.2		14.7		20.5		2.6	
Wearing spectacles or contact lenses										
Yes (*n* = 755)	15.6	**<0.001**	22.9	**0.005**	15.0	**0.03**	23.0	**0.04**	7.5	**<0.001**
No (*n* = 335)	6.0		15.5		10.1		17.6		2.1	
Eye condition diagnosed by a doctor							21.4			
Yes (*n* = 386)	22.0	**<0.001**	25.4	**0.004**	15.3	0.2	25.4	**0.02**	11.7	**<0.001**
No (*n* = 704)	7.5		18.0		12.5		19.2		2.7	

Statistically significant values are bolded.

## Discussion

This nationwide cross-sectional study provided evidence on public knowledge and habits related to dietary supplement use for eye health among adults in Poland. Only one-tenth of respondents declared good or very good self-reported knowledge (10.2%) of the impact of diet and lifestyle on vision and eye health, but over one-fourth (27.9%) reported using dietary supplements for eye health, indicating that supplementation is relatively common despite limited awareness of evidence based recommendations. Supplement use may be influenced by eye symptom experience, personal beliefs, and external influences, in addition to evidence-based recommendations (22, 23). Active occupational status (employed or self-employed individuals) as well as wearing spectacles or contact lenses and history of eye conditions diagnosed by a doctor were significantly associated with higher odds of dietary supplementation to support eye health and vision. This observation may indicate that individuals with eye conditions or vision problems are more likely to report dietary supplement use.

As this study was designed as a population-based cross-sectional survey, the findings should primarily be interpreted in the context of public awareness, self-reported behaviors, and patterns of dietary supplement use rather than clinical effectiveness of nutritional interventions.

This study was particularly focused on eye health and nutrition and revealed that most of the adults in Poland present low self-reported knowledge on the impact of diet on eye health. Data from previously published population-based studies in Poland showed that nutritional knowledge is associated with sociodemographic factors, particularly gender and education level (37). In general, females and individuals with higher educational presented higher nutritional knowledge (37). However, although greater knowledge has been linked to better diet quality, it does not necessarily translate into optimal health behaviors (37). This may partly explain the patterns observed in the present study, where relatively frequent use of dietary supplements coexists with limited knowledge regarding the role of diet and lifestyle in eye health. In this study, females were also identified as a group with higher self-reported knowledge on diet and eye health. Moreover, in this study place of residence (living in the smallest or the largest cities) was also associated with self-reported knowledge on the impact of diet on eye health and vision. This observation may reflect differences in access to health-related information and healthcare services between urban and rural populations. Residents of large cities may have greater exposure to public health campaigns, specialist healthcare services, and digital health information sources. In contrast, respondents from the smallest towns may present stronger engagement in local health initiatives or more frequent use of primary healthcare services, which may influence self-reported knowledge related to diet and eye health. This observation confirms the impact of sociodemographic factors on Poles knowledge related to nutrition and supplementation (30, 31, 37). Although selected nutrients have been associated with eye health in previous studies, public awareness of evidence-based nutritional recommendations for vision support appears to remain limited (14–18). It should also be noted that the direction of the observed associations cannot be determined due to the cross-sectional design of the study. Individuals with eye conditions or prior experience with dietary supplements may perceive themselves as more knowledgeable regarding diet and eye health.

Dietary supplement use for eye health was reported by a considerable proportion of respondents (27.9%), although most individuals used supplements for relatively short periods (≤6 months). This pattern may indicate that supplementation is often undertaken in response to transient symptoms rather than as part of a long-term preventive strategy. The prevalence of dietary supplement use observed in this study (27.9%) appears lower than estimates reported in developed countries, where approximately 30–50% of adults use such products (38). However, this study was focused specifically on dietary supplements for eye health.

Among supplement users, the most frequently reported reasons included personal preventive decisions, recommendations from ophthalmologists, and the presence of symptoms such as eye fatigue or dryness. A large proportion of adults who used dietary supplements for eye health and vision reported making independent decisions regarding supplementation. This observation is in line with previously published data on motivations toward dietary supplementations in Poland. This observation is in line with findings from a representative cross-sectional survey conducted in December 2024 among 5,006 adults aged 18–64 years in Poland, which showed that among individuals who had used dietary supplements in the preceding 3 months, only 11.4% reported that all supplements had been prescribed by a doctor, while 22.7% reported that only some of them had been physician-recommended (31). These findings support the view that dietary supplement use in Poland is largely driven by self-directed practices rather than exclusively by medical advice.

In this study, the use of dietary supplements for eye health was strongly associated with the presence of eye conditions and wearing spectacles or contact lenses. Individuals with diagnosed eye conditions were more than three times as likely to report supplement use, suggesting that supplementation may be more strongly related to perceived health needs, symptom experience, or previous healthcare interactions rather than preventive actions directly resulting from knowledge alone. Similarly, those wearing spectacles or contact lenses were significantly more likely to use supplements, which may reflect increased awareness of visual function or greater concern about eye health. These findings suggest that ophthalmologists may play important role in recommendations of dietary supplementation for patients with eye conditions or vision impairment. This is in line with scientific papers on effectiveness of dietary supplements for eye health, that is mostly limited to those at higher risk of eye conditions like AMD (12–18, 20–24). The association observed for occupationally active individuals suggests that lifestyle-related factors, such as higher screen exposure or greater financial capacity, may also contribute to supplementation behaviors. Moreover, occupationally active individuals may have higher economic status and financial resources to buy dietary supplements.

The composition of dietary supplements used by respondents reflects general market trends, with antioxidant vitamins and omega-3 fatty acids being the most commonly reported ingredients (19). These products are promoted for general health benefits that may lead to its higher consumption observed in this study. Lutein and zeaxanthin were consumed less frequently despite better evidence for their positive impact on eye health (15, 16, 20). The relatively low use of multicomponent formulations dedicated to eye health (5.9%) suggests that consumers may preferentially select more familiar or broadly marketed products rather than specialized, indication-specific formulations. Moreover, the observed association with self-reported financial status may indicate that economic factors also influence the use of specialized multicomponent formulations, which are often more expensive than single-ingredient products.

Importantly, the physiological role of selected micronutrients in maintaining normal visual function should be distinguished from evidence regarding dietary supplementation for prevention or treatment of eye diseases. Although AREDS and AREDS2 studies demonstrated benefits of selected antioxidant formulations in slowing progression of intermediate AMD in high-risk patients, evidence supporting routine supplementation in the general population remains limited (39, 40). Therefore, dietary supplements for eye health should be used in an evidence-based and medically supervised manner.

The observed association between the use of glasses or contact lenses and higher dietary supplement use may reflect a belief that supplementation can improve visual function or refractive conditions. However, current evidence supporting the effectiveness of supplements in refractive errors remains limited. This may have practical public health implications, particularly if similar beliefs are transferred to children with refractive errors. Therefore, individuals with refractive correction could represent a potential target group for evidence-based educational and awareness campaigns regarding appropriate supplement use and eye health.

The observed associations may also be influenced by unmeasured factors, including general health awareness, access to healthcare services, previous healthcare experiences, or exposure to marketing and non-professional information sources related to dietary supplements.

Importantly, contemporary nutritional ophthalmology increasingly emphasizes overall dietary patterns rather than isolated nutrient supplementation alone. Recent evidence suggests that adherence to healthy dietary models, particularly the Mediterranean diet, may positively influence ocular surface health through anti-inflammatory and antioxidant mechanisms. Ahmadzadeh et al. highlighted the potential role of Mediterranean diet components in dry eye disease prevention and symptom reduction, emphasizing the importance of comprehensive dietary quality alongside evidence-based supplementation strategies (41).

### Practical implications

Findings from this study highlight the need to strengthen public awareness of the role of diet in eye health and to promote the evidence-based use of dietary supplements. Healthcare professionals should play a more active role in providing guidance on dietary supplement use based on scientific evidence, especially when advising patients at higher risk of eye conditions. These actions are needed to reduce reliance on self-medication and non-professional or non-evidence-based sources of information on the role of dietary supplementation in supporting eye health and vision. Public health strategies should focus on improving public awareness and evidence-based knowledge regarding diet and eye health. Moreover, strengthening regulatory oversight of the dietary supplement market may be considered to protect consumers against misleading marketing claims regarding the impact of supplements on health and disease prevention.

### Limitations

This study has several limitations, mostly related to its cross-sectional design. As both knowledge assessment and dietary supplement use were based on self-reported responses, the findings may be affected by recall bias, social desirability bias, and subjective interpretation of personal knowledge levels. Therefore, causal relationships and reverse causality between self-reported knowledge, eye conditions, and dietary supplement use cannot be excluded. Participation in the online research panel was voluntary, which may have introduced a certain degree of selection bias typical for web-based surveys. Nevertheless, quota sampling based on demographic characteristics was applied to improve sample representativeness. Daily habits like eating behaviors and lifestyle were not verified during in-person observation. Knowledge of the impact of diet and lifestyle on vision and eye health was self-declared. Self-prepared questionnaire was used, as there were no validated questionnaires that can be adapted in polish settings. Nevertheless, pilot study was conducted. Participants were asked about consumption of only 5 major ingredients of dietary supplements and vitamin–mineral preparations, listed in scientific literature as those with potential impact on eye health. The CAWI method may introduce selection bias by excluding individuals without internet access, although this group represents less than 4% of households in Poland. Moreover, individuals with limited digital literacy, particularly the oldest adults, may have been underrepresented in the study population. This is especially important in the context of eye health research, as older individuals are at higher risk of chronic ophthalmological conditions and visual impairment. Therefore, caution is warranted when generalizing the findings to the oldest population groups. Additionally, no detailed data on dosage or precise clinical verification of eye conditions were collected, which may affect the accuracy of the findings.

## 5 Conclusion

Dietary supplement use for eye health was reported by a considerable proportion of respondents, while most participants declared low or moderate self-reported knowledge regarding the impact of diet on eye health and vision. Higher levels of knowledge were more frequently observed among women and individuals living in urban areas. The findings suggest that dietary supplement use is associated with multiple factors beyond self-reported knowledge on diet and eye health. These findings highlight the need for improved public education on nutrition and eye health, as well as a stronger role of healthcare professionals in guiding appropriate, evidence-based supplement use.

## Data Availability

The raw data supporting the conclusions of this article will be made available by the authors, without undue reservation.

## References

[1] Lane R. Of sight, and insight. Lancet. (2023) 401:97. doi: 10.1016/S0140-6736(22)02589-2 37235452

[2] Hutmacher F. Why is there so much more research on vision than on any other sensory modality? Front Psychol. (2019) 10:2246. doi: 10.3389/fpsyg.2019.02246, 31636589 PMC6787282

[3] World Health Organization. Blindness and visual Impairment. Geneva: WHO (2024). Available online at: https://www.who.int/news-room/fact-sheets/detail/blindness-and-visual-impairment (Accessed April 21, 2026).

[4] Zheng M Yin T Jiang Z Li X Fang B Pan M . Global, regional and national burden and trends of sense organ diseases from 1990 to 2021. BMJ Open. (2025) 15:e104038. doi: 10.1136/bmjopen-2025-104038, 41309476 PMC12666223

[5] Zhao C Ding Q Yang Z. Burdens and trends of blindness and vision loss among those aged ≥55 years. Eur J Ophthalmol. (2024) 34:1852–64. doi: 10.1177/11206721241238878, 38454852

[6] Zhang Q Jiang Y Deng C Wang J. Effects and potential mechanisms of exercise and physical activity on eye health and ocular diseases. Front Med (Lausanne). (2024) 11:1353624. doi: 10.3389/fmed.2024.1353624, 38585147 PMC10995365

[7] Li K Tang J Zhang Z Li X Zheng Y Jiang D . Global burden and cross-country inequalities of age-related eye diseases from 1990 to 2021: a comprehensive analysis of temporal trends and socioeconomic disparities. Eye Vis (Lond). (2026) 13:4. doi: 10.1186/s40662-026-00473-5, 41620804 PMC12861071

[8] GBD 2021 Global AMD Collaborators. Global burden of vision impairment due to AMD. Lancet Glob Health. (2025) 13:e1175–90. doi: 10.1016/S2214-109X(25)00143-3 40580986 PMC12208786

[9] Lawrenson JG Downie LE. Nutrition and eye health. Nutrients. (2019) 11:2123. doi: 10.3390/nu11092123, 31489894 PMC6771137

[10] Bogdănici CG Bogdănici CM Pavel IA Ganea CV Donica VC Cărăușu EM. Associations between nutritional factors, obesity and ocular diseases: a narrative literature review. Nutrients. (2025) 17:3798. doi: 10.3390/nu17233798, 41374087 PMC12694112

[11] Evans JR Lawrenson JG. Antioxidant vitamin and mineral supplements for slowing the progression of age-related macular degeneration. Cochrane Database Syst Rev. (2023) 9:CD000254. doi: 10.1002/14651858.CD000254.pub5, 37702300 PMC10498493

[12] Davey PG Ranganathan A. Editorial: Feast your eyes: diet and nutrition for optimal eye health. Front Nutr. (2025) 12:1579901. doi: 10.3389/fnut.2025.1579901, 40104816 PMC11913715

[13] Massoudi S Azizi-Soleiman F Yazdi M Meghdadi Esfahani M Heidari-Beni M Kelishadi R. The association between macronutrients intake and myopia risk: a systematic review and meta-analysis. BMC Ophthalmol. (2024) 24:472. doi: 10.1186/s12886-024-03738-6, 39472829 PMC11523676

[14] Cong Y Zhang Y Han Y Wu Y Wang D Zhang B. Recommendations for nutritional supplements for dry eye disease: current advances. Front Pharmacol. (2024) 15:1388787. doi: 10.3389/fphar.2024.1388787, 38873421 PMC11169594

[15] Chew EY Clemons TE Agrón E Domalpally A Keenan TDL Vitale S . Long-term outcomes of adding lutein/zeaxanthin and ω-3 fatty acids to the AREDS supplements on age-related macular degeneration progression: aREDS2 Report 28. JAMA Ophthalmol. (2022) 140:692–8. doi: 10.1001/jamaophthalmol.2022.1640, 35653117 PMC9164119

[16] Mrowicka M Mrowicki J Kucharska E Majsterek I. Lutein and zeaxanthin and their roles in age-related macular degeneration-neurodegenerative disease. Nutrients. (2022) 14:827. doi: 10.3390/nu14040827, 35215476 PMC8874683

[17] Wang WX Ko ML. Efficacy of Omega-3 intake in managing dry eye disease: a systematic review and meta-analysis of randomized controlled trials. J Clin Med. (2023) 12:7026. doi: 10.3390/jcm12227026, 38002640 PMC10672334

[18] Medori MC Naureen Z Dhuli K Placidi G Falsini B Bertelli M. Dietary supplements in retinal diseases, glaucoma, and other ocular conditions. J Prev Med Hyg. (2022) 63:E189–99. doi: 10.15167/2421-4248/jpmh2022.63.2S3.2760, 36479474 PMC9710404

[19] Global Market Insights. Eye health supplements market. (2024). Available online at: https://www.gminsights.com/industry-analysis/eye-health-supplements-market (Accessed April 21, 2026)

[20] Lopresti AL Smith SJ. The effects of lutein/ zeaxanthin (Lute-gen®) on eye health, eye strain, sleep quality, and attention in high electronic screen users: a randomized, double-blind, placebo-controlled study. Front Nutr. (2025) 12:1522302. doi: 10.3389/fnut.2025.1522302, 39963662 PMC11830589

[21] Downie LE Ng SM Lindsley KB Akpek EK. Omega-3 and omega-6 polyunsaturated fatty acids for dry eye disease. Cochrane Database Syst Rev. (2019) 12:CD011016. doi: 10.1002/14651858.CD011016.pub2, 31847055 PMC6917524

[22] Rautiainen S Manson JE Lichtenstein AH Sesso HD. Dietary supplements and disease prevention - a global overview. Nat Rev Endocrinol. (2016) 12:407–20. doi: 10.1038/nrendo.2016.54 27150288

[23] Antonio J Antonio B Aragon A Bustillo E Candow D Collins R . Common questions and misconceptions about dietary supplements and the industry - What does science and the law really say? J Int Soc Sports Nutr. (2025) 22:2534128. doi: 10.1080/15502783.2025.2534128, 40662344 PMC12265102

[24] English LK Raghavan R Obbagy JE Callahan EH Fultz AK Nevins JEH . Dietary patterns and health: insights from NESR systematic reviews to inform the Dietary Guidelines for Americans. J Nutr Educ Behav. (2024) 56:75–87. doi: 10.1016/j.jneb.2023.10.001 38185492

[25] Janzik R Geppert J Müller P Notz I Obstfeld H Roth B . Exploring motivations, information behavior, perceptions, and intentions among dietary supplement users: a cross-sectional survey study in Germany. Front Nutr. (2025) 12:1663562. doi: 10.3389/fnut.2025.1663562, 41127098 PMC12537374

[26] Strocka J Religioni U Plagens-Rotman K Drab A Merks P Kaźmierczak J . Knowledge and practices regarding dietary supplements among healthcare professionals in Poland. Nutrients. (2024) 16:3691. doi: 10.3390/nu16213691, 39519524 PMC11547466

[27] Broniecka A Sarachman A Zagrodna A Książek A. Dietary supplement use and knowledge among athletes: prevalence, compliance with AIS classification, and awareness of certification programs. J Int Soc Sports Nutr. (2025) 22:2496450. doi: 10.1080/15502783.2025.2496450, 40263114 PMC12016268

[28] Zovi A Vitiello A Sabbatucci M Musazzi UM Sagratini G Cifani C . Food supplement regulation. J Diet Suppl. (2025) 22:25–40. doi: 10.1080/19390211.2024.2389397 39221704

[29] Djaoudene O Romano A Bradai YD Zebiri F Ouchene A Yousfi Y . A Global overview of dietary supplements: regulation, market trends, usage during the COVID-19 pandemic, and health effects. Nutrients. (2023) 15:3320. doi: 10.3390/nu15153320, 37571258 PMC10421343

[30] Strocka J Religioni U Pinkas J. Food supplements market in Poland and the European Union – regulations and consumption of food supplements in the pre-COVID and COVID era. Fam Med Prim Care Rev. (2025) 27:347–51. doi: 10.5114/fmpcr.2025.153097

[31] Sierpiński R Jankowski M Raciborski F Kamińska A. Supplement use in Poland. Front Nutr. (2025) 12:1724264. doi: 10.3389/fnut.2025.1724264 41477317 PMC12751291

[32] Wierzejska RE. Dietary supplements—for whom? Int J Environ Res Public Health. (2021) 18:8897. doi: 10.3390/ijerph18178897, 34501487 PMC8431076

[33] Nationwide Research Panel Ariadna. (2026). Available online at: https://panelariadna.com (Accessed April 20, 2026).

[34] Statistics Poland. Demographic Yearbook of Poland 2024 (2025). Available online at: https://stat.gov.pl (Accessed April 20, 2026).

[35] Olearczyk A Sękowski K Jankowski M Moczeniat G Kamińska A Grudziąż-Sękowska J. Prevalence and drivers of missed healthcare appointments in Poland: insights from a 2025 survey. Med Sci Monit. (2026) 32:e951944. doi: 10.12659/MSM.951944, 41764069 PMC12961910

[36] Kamińska A Pinkas J Jankowski M. Factors associated with the frequency of eye examinations among adults in Poland - a nationwide cross-sectional survey, December 2022. Ann Agric Environ Med. (2023) 30:287–95. doi: 10.26444/aaem/159152, 37387379

[37] Kucharska A Sińska BI Panczyk M Samel-Kowalik P Raciborski F Czerwonogrodzka-Senczyna A . Nutritional knowledge, sociodemographic, and lifestyle factors as determinants of diet quality - a Polish population-based study. Front Public Health. (2025) 13:1613598. doi: 10.3389/fpubh.2025.1613598, 40917426 PMC12408304

[38] Iłowiecka K Maślej M Czajka M Pawłowski A Więckowski P Styk T . Lifestyle, eating habits, and health behaviors among dietary supplement users in three European countries. Front Public Health. (2022) 10:892233. doi: 10.3389/fpubh.2022.892233, 35719650 PMC9198248

[39] Evans JR Lawrenson JG. Antioxidant vitamin and mineral supplements for preventing age-related macular degeneration. Cochrane Database Syst Rev. (2017) 2017:CD000253. doi: 10.1002/14651858.CD000253.pub4, 28756617 PMC6483250

[40] European Food Safety Authority. EFSA publications [Internet]. (2026). Available online at: https://efsa.onlinelibrary.wiley.com/ (Accessed May 12, 2026).

[41] Ahmadzadeh O Ghafouri D Shadnoush M Samani P Mirzaei A Javid Z . Mediterranean diet components and dry eye disease: current evidence and mechanistic insights. Clin Nutr Open Sci. (2026) 66:100625. doi: 10.1016/j.nutos.2026.100625

